# Supplementary material for: Male genital lobe morphology affects the chance to copulate in Drosophila pachea
Source: BMC Ecol Evol. 2021 Feb 11;21:23. doi: 10.1186/s12862-021-01759-z (PMC7877081; doi:10.1186/s12862-021-01759-z)
Supplement: Supplementary file 1 — Additional file 1. Additional Figures and Tables, Figure S1. Camera device for movie recording. Figure S2. Courtship and copulation duration in competition mating experiments. Figure S3. Failed copulation attempts compared to female age. Figure S4. Courtship duration increases with female age. Figure S5. Pairwise lobe length differences of males used in the competition mating experiment. Figure S6. Pearson correlations of male specific predictor variables. Table S1. Drosophila pachea resources. Table S2. Genital lobe lengths in D. pachea stocks. Table S3. Courtship duration, male licking behavior and copulation duration in trials where both males courted the female. Table S4. Competition mating trials used for courtship analysis. Tables S5–S12. Bradley Terry Model Fit. [file 12862_2021_1759_MOESM1_ESM.pdf]

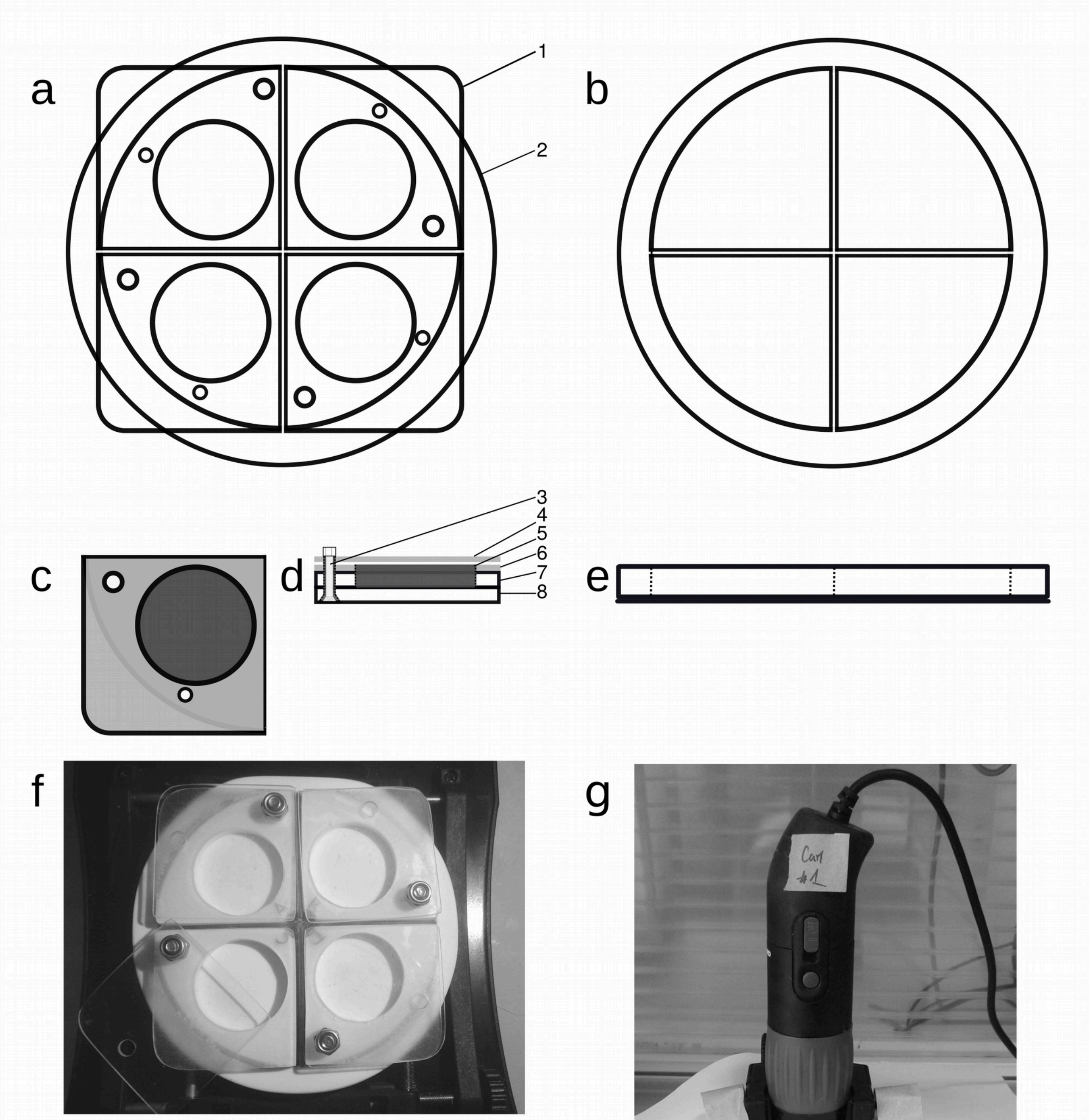

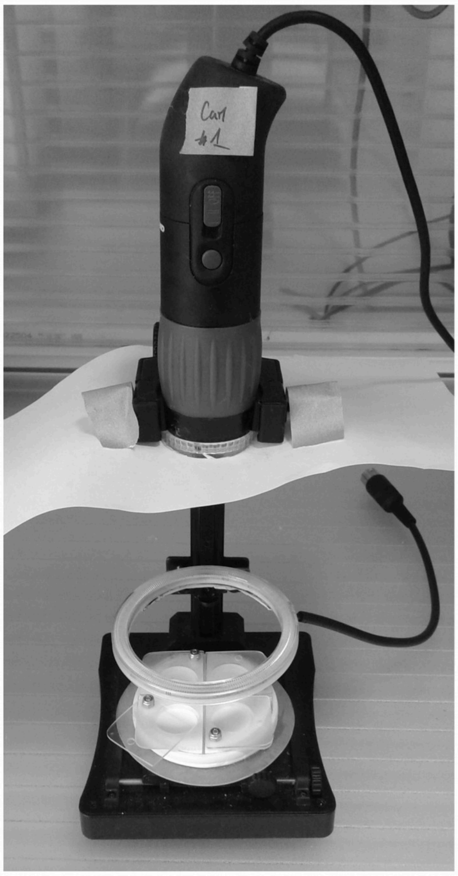

Figure S1: Camera device for movie recording. a) Schematic drawing of 4 mating cells (1) arranged on a round support (2); b) The round support has an inner diameter of 62 mm and is cross-divided (1 mm plexiglas) to hold four individual mating cells; c) the mating cell is a 30 mm quadrant (top view). A 3 mm screw (3) holds a transparent top cover (4), which has 3 mm bore for the screw and a 2 mm bore to introduce flies. d) The mating cell in lateral view shows the layer construction: It is assembled with a 3 mm screw (3) that holds the 1 mm thick transparent Plexiglas cover (4) on top of a 7 mm thick plastic unit with the cylindrical 20 mm x 4 mm inside of the mating cell (5). It consists of a 1 mm transparent Plexiglass layer (6) that serves as a handle and has an inner bore of 20 mm, a 3 mm thick white Plexiglas layer (7) with the inner bore, and a 3 mm thick white Plexiglas bottom layer (8). e) The round mating cell support in lateral view. It consists of a Plexiglas ring of 5 mm, an outer diameter of 70 mm and an inner diameter of 60 mm. It is glued to a 1 mm thick Plexiglas bottom layer. Dashed lines indicate the inner edge of the Plexiglas ring and the 1 mm cross division. f) The assembled mating cell device with 4 mating cells and the support mounted on a microscope stand (191348, Conrad). g) The mating cell device mounted on the microscope stand and equipped with a LED white light 3-12 V, 8-cm illumination ring (EB-AE-COB-Cover, YM E-Bright) and a camera (MIRAZOOM MZ902, OWL).

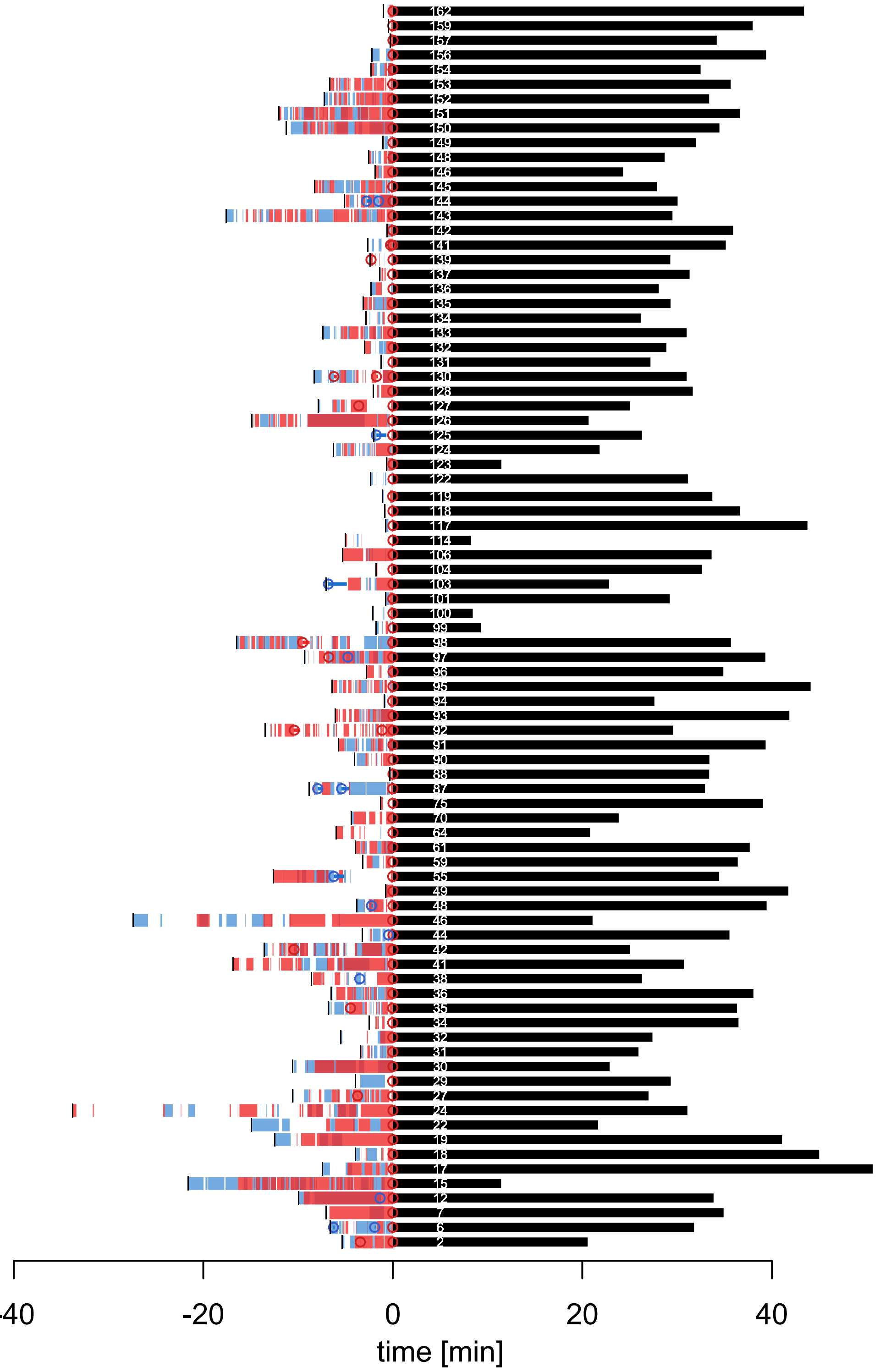

Figure S2: Courtship and copulation duration in competition mating experiments. The black dash indicates courtship start, segments indicate licking periods, open circles mark mounting events and horizontal bars indicate the durations of failed mounting attempts of the copulating male (red) and the non-copulating male (blue). The black bar indicates copulation duration and the number refers to the trial (mating ID), described in Additional datafile 3 and Additional datafile 4.

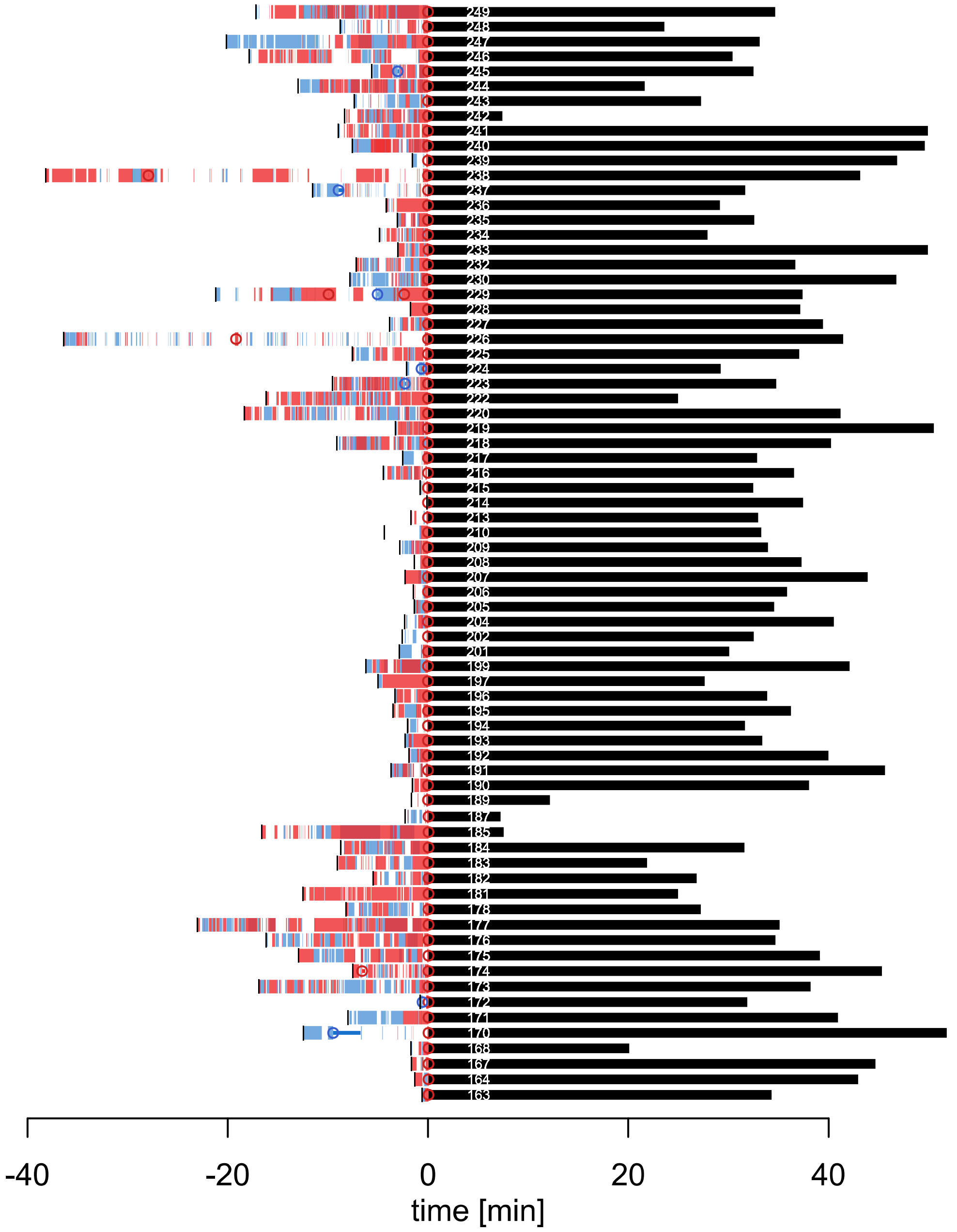

Figure S2 (continued): Courtship and copulation duration in competition mating experiments. The black dash indicates courtship start, segments indicate licking periods, open circles mark mounting events and horizontal bars indicate the durations of failed mounting attempts of the copulating male (red) and the non-copulating male (blue). The black bar indicates copulation duration and the number refers to the trial (mating ID), described in Additional datafile 3 and Additional datafile 4.

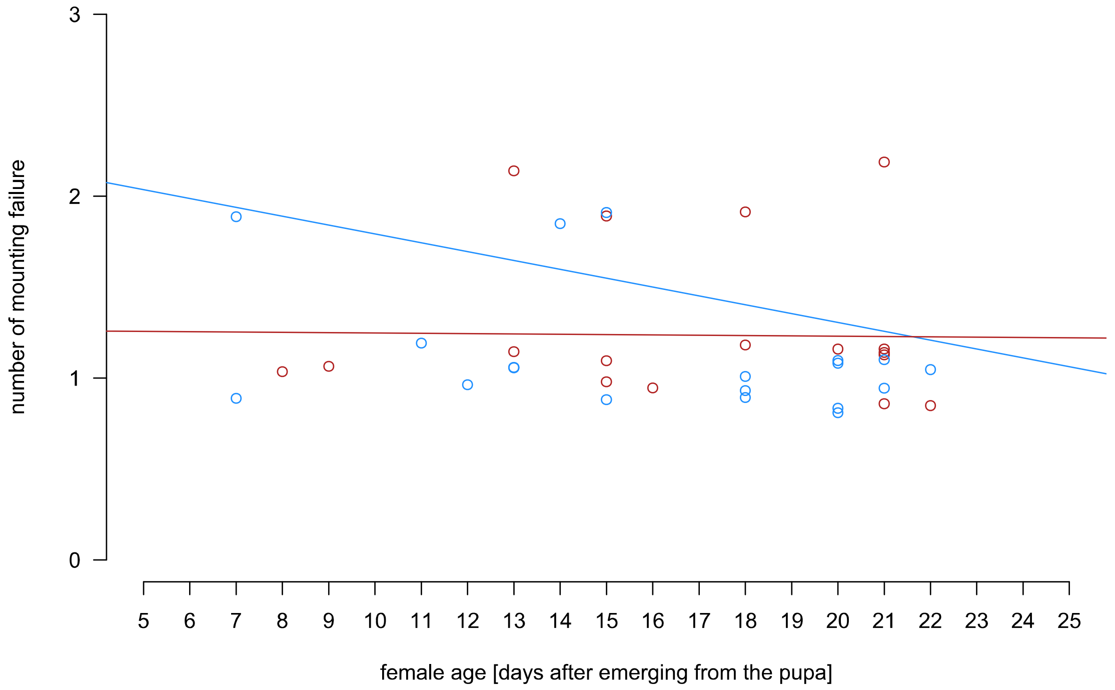

Figure S3: Failed copulation attempts compared to female age. Failed copulation attempts of the non-copulating male (blue circles) and the copulating male (red circles) with linear regression lines (non-copulating males: $r^2 = 0.019694$, $y = -0.04872x + 2.2795$, blue line, and copulating males: $r^2 = 0.00029967$, $y = -0.001731x + 1.2643$, red line).

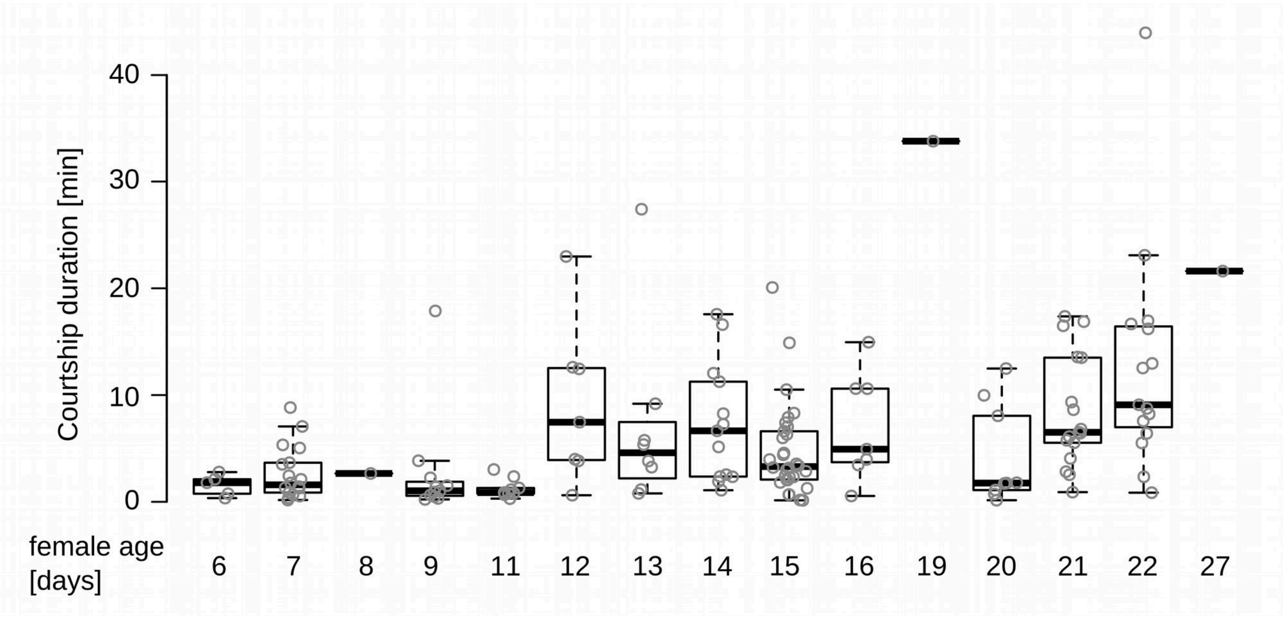

Figure S4: Courtship duration increases with female age. The average courtship duration is increased in females that are older than 11 days after hatching from the pupa (Mann-Whitney test, W = 1525.5, *N females younger than 12 days / N females older than 11 days = 52 / 161, P* = 5.811×10^{-12}). Pearson correlation of courtship duration and female age: 0.41, P= 4.68×10^{-6}.

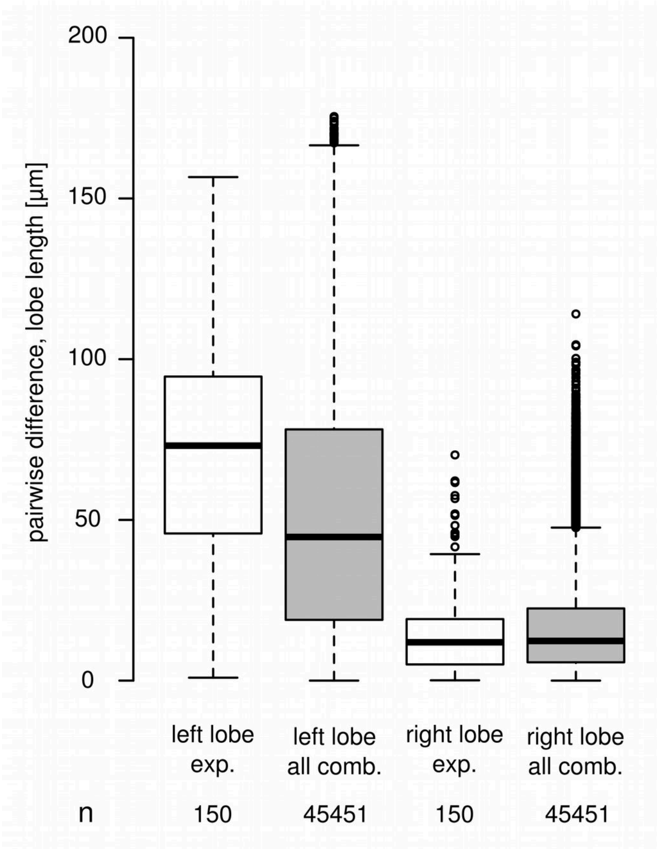

Figure S5: Pairwise lobe length differences of males used in the competition mating experiments. The lobe length pairwise length differences of selected pairs in our mating competition experiments (exp., white boxplots) compared to lobe length differences of the same males but randomly compared (random comp., grey boxplots).

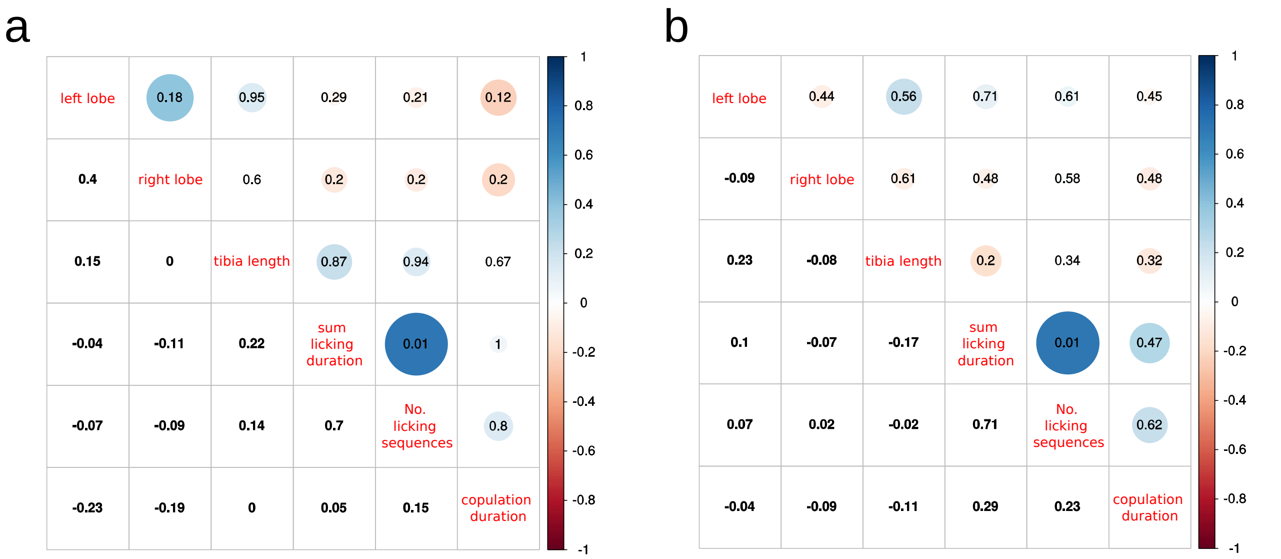

Figure S6: Pearson correlations of male specific predictor variables. a) dataset with unmodified males (92 trials), **b)** dataset with lobe-modified males (53 trials). Variable names are listed in the square diagonal, coefficients are in the lower and P-values in the upper half of the square. Circle colors indicate the coefficient according to the scale bars on the right and circle size is proportional to the P-value.

Table S1: *Drosophila pachea* Resources

source	stock number	collection locality	collection year
Drosophila Species Stock Center	15090-1698.01	USA<br>Arizona<br>Organ Pipe Cactus National Monument	1997
Drosophila Species Stock Center	15090-1698.02	Mexico<br>Sonora<br>Bahia de Kino	1996

Table S2: Genital lobe lengths in *D. pachea* stocks. †Confidence interval

stock	left lobe [µm], median (95% CI†)	right lobe [µm] median, (95% CI†)	n
15090-1698.01	206.7 (169.7 - 215.4)	147.6 (145.4 - 153.1)	50
15090-1698.02	204.7 (190.5 - 209.2)	145.4 (138.6 - 146.4)	50
selection stock	186.1 (171.8 - 185.0)	142.2 (138.7 - 144.2)	99

Table S3: Courtship duration, male licking behavior and copulation duration in trials where both males courted the female

female	male	n	total courtship duration [min]		sum licking duration [min]		No. licking sequences		copulation duration [min]	
			median	range	median	range	median	range	median	range
15090-1698.01	non-copulating sel. lobe length	47	3.23	0.30-33.80	0.63	0.02-7.68	4	1-22	---	---
	copulating sel. lobe length	47			1.00	0.10-12.53	3	1-29	32.84	10.13-49.21
15090-1698.02	non-copulating sel. lobe length	57	6.3	0.74-23.10	1.58	0.04-14.24	5	1-36	---	---
	copulating sel. lobe length	57			2.80	0.14-14.58	5	1-35	27.7	2.72-50.21
15090-1698.01	non-copulating modif. lobe length	54	7.2	0.12-38.18	1.28	0.02-12.22	7	1-64	---	---
	copulating modif. lobe length	54			1.84	0.10-14.92	6.5	1-34	33.05	3.45-49.23

Table S4: Competition mating trials used for courtship analysis. †Experiment excluded when: At least one fly escaped, died, or was injured, the movie recording or data storage failed, or genitalia dissection failure (Additional datafile 3).

female stock	male stock	Lobe surgery	No. Experiments					
			total	included	excluded: no copulation / courtship	excluded: other†	male courtship: one	male courtship: both
15090-1698.01	selection stock	No	98	76	16 / 4	2	25	51
15090-1698.02	selection stock	No	89	76	4 / 0	9	16	60
15090-1698.01	selection stock	Yes	62	61	1 / 0	0	7	54

Table S5: Bradley-Terry Model fit for 92 trials with non lobe-modified males, evaluation of male-specific predictor variables with exclusion of number of licking sequences. Model: copulation success ~ left lobe length + right lobe length + tibia length + failed copulation attempt + sum licking duration, null deviance: 127.539 on 92 degrees of freedom, residual deviance: 94.643 on 86 degrees of freedom.

variable	estimate	standard error	z-value	P (>|z|)
left lobe length	0.007358	0.003509	2.097	**0.035997**
right lobe length	-0.016259	0.015030	-1.082	0.279366
tibia length	0.011875	0.007138	1.664	0.096199
failed copulation attempt	-0.347733	0.554350	-0.627	0.530476
sum licking duration	0.769157	0.221818	3.468	**0.000525**

Table S6: Bradley-Terry Model fit for 92 trials with non lobe-modified males, evaluation of male-specific predictor variables with exclusion of sum licking duration. Model: copulation success ~ left lobe length + right lobe length + tibia length + failed copulation attempt + number of licking sequences, null deviance: 127.539 on 92 degrees of freedom, residual deviance: 114.96 on 87 degrees of freedom.

variable	estimate	standard error	z-value	P (>|z|)
left lobe length	0.007008	0.003175	2.207	**0.0273**
right lobe length	-0.005840	0.012564	-0.465	0.6420
tibia length	0.012172	0.006625	1.837	0.0662
failed copulation attempt	-0.071513	0.448564	-0.159	0.8733
number of licking sequences	0.093730	0.060826	1.541	0.1233

Table S7: Bradley-Terry Model fit for 92 trials with non lobe-modified males, evaluation of interactions of male-specific variables with female stock. Model: copulation success ~ (left lobe length + right lobe length + tibia length + failed copulation attempt + sum licking duration) * female stock, null deviance: 127.539 on 92 degrees of freedom, residual deviance: 87.62 on 82 degrees of freedom.

variable	estimate	standard error	z-value	P (>|z|)
left lobe length	0.0070509	0.0053873	1.309	0.1906
right lobe length	-0.0164323	0.0221690	-0.741	0.4586
tibia length	0.0002137	0.0122500	0.017	0.9861
failed copulation attempt	-0.3510129	0.6557440	-0.535	0.5924
sum licking duration	0.4372115	0.2212514	1.976	**0.0481**
female stock (1698.02)	NA	NA	NA	NA
left lobe length * female stock (1698.02)	0.0057967	0.0086074	0.673	0.5007
right lobe length * female stock (1698.02)	-0.0144092	0.0346259	-0.416	0.6773
tibia length * female stock (1698.02)	0.0235552	0.0166775	1.412	0.1578
failed copulation attempt * female stock (1698.02)	0.0355114	1.2123092	0.029	0.9766
sum licking duration * female stock (1698.02)	1.0801333	0.5623220	1.921	0.0548

Table S8: Bradley-Terry Model fit for 92 trials with non lobe-modified males, evaluation of interactions of male-specific variables with courtship duration. Sum licking duration and number of licking sequences were excluded from the model because they are redundant with courtship duration. Model: copulation success ~ (left lobe length + right lobe length + tibia length + failed copulation attempt) * courtship duration, null deviance: 127.539 on 92 degrees of freedom, residual deviance: 115.21 on 84 degrees of freedom.

variable	estimate	standard error	z-value	P (>|z|)
left lobe length	0.0046696	0.0052433	0.891	0.373
right lobe length	-0.0038326	0.0191598	-0.200	0.841
tibia length	0.0059164	0.0096083	0.616	0.538
failed copulation attempt	-1.1040192	1.0071044	-1.096	0.273
courtship duration	NA	NA	NA	NA
left lobe length * courtship duration	0.0003350	0.0006249	0.536	0.592
right lobe length * courtship duration	-0.0005112	0.0021788	-0.235	0.814
tibia length * courtship duration	0.0008349	0.0012409	0.673	0.501
failed copulation attempt* courtship duration	0.1451978	0.1150052	1.263	0.207

Table S9: Bradley-Terry Model fit for 92 trials with non lobe-modified males, evaluation of interactions of male-specific variables with female age. Model: copulation success ~ (left lobe length + right lobe length + tibia length + failed copulation attempt + sum licking duration) * female age, null deviance: 127.539 on 92 degrees of freedom, residual deviance: 87.95 on 82 degrees of freedom.

variable	estimate	standard error	z-value	P (>|z|)
left lobe length	1.520e-02	1.274e-02	1.193	0.2327
right lobe length	-5.233e-02	5.401e-02	-0.969	0.3326
tibia length	1.136e-02	2.200e-02	0.516	0.6057
failed copulation attempt	-4.394e+00	2.128e+00	-2.065	**0.0389**
sum licking duration	1.190e+00	9.719e-01	1.225	0.2207
female age	NA	NA	NA	NA
left lobe length * female age	-4.739e-04	7.608e-04	-0.623	0.5334
right lobe length * female age	2.102e-03	3.389e-03	0.620	0.5350
tibia length * female age	5.203e-05	1.448e-03	0.036	0.9713
failed copulation attempt * female age	2.773e-01	1.322e-01	2.098	**0.0359**
sum licking duration * female age	-1.624e-02	5.514e-02	-0.294	0.7684

Table S10: Bradley–Terry (BT) model examining the effects of left lobe length, sum licking duration and failed mounting attempts on copulation success, in 92 trials with unmodified males. The main effect for "female age" cannot be estimated in the BT model because it is specific to each trial and not to each male. Bolded estimates are significant. Model: copulation success ~ sum licking duration + left lobe length + failed mounting attempts * female age, null deviance: 127.539 on 92 degrees of freedom, residual deviance: 92.813 on 88 degrees of freedom. Influential trials 29 and 141 were detected to affect model estimates.

variable	estimate	standard error	z-value	P (>|z|)
sum licking duration	0.867081	0.244225	3.550	**0.000385**
left lobe length	0.006994	0.003450	2.027	**0.042637**
failed copulation attempt	-3.7406217	1.783746	-2.097	**0.035989**
female age	NA	NA	NA	NA
failed mounting attempts x female age	0.245507	0.117685	2.086	**0.036966**

Table S11: Bradley–Terry (BT) model examining the effects of left lobe length, right lobe length, number of licking sequences, tibia length and failed mounting attempts on copulation success, in 53 trials with lobe-modified males.Model: copulation success ~ left lobe length + right lobe length + tibia length + failed copulation attempt + number of licking sequences, null deviance: 73.474 on 53 degrees of freedom, residual deviance: 69.250 on 48 degrees of freedom.

variable	estimate	standard error	z-value	P (>|z|)
left lobe length	0.0088769	0.0048547	1.829	0.0675
right lobe length	0.0098511	0.0229071	0.430	0.6672
tibia length	-0.0007568	0.0079174	-0.096	0.9239
failed copulation attempt	0.0611166	0.8192932	0.075	0.9405
number of licking sequences	-0.0340544	0.0412683	-0.825	0.4093

Table S12: Bradley–Terry (BT) model examining the effects of left lobe length, right lobe length, sum licking duration, tibia length and failed mounting attempts on copulation success, in 53 trials with lobe-modified males. Model: copulation success ~ left lobe length + right lobe length + tibia length + failed copulation attempt + sum licking duration, null deviance: 73.474 on 53 degrees of freedom, residual deviance: 67.020 on 48 degrees of freedom.

variable	estimate	standard error	z-value	P (>|z|)
left lobe length	0.0067083	0.0049313	1.360	0.174
right lobe length	0.0064819	0.0234845	0.276	0.783
tibia length	0.0009478	0.0079822	0.119	0.905
failed copulation attempt	-0.3486010	0.9209974	-0.379	0.705
sum licking duration	0.2443697	0.1641949	1.488	0.137
